# Computational investigation of the sequence context of arginine/glycine-rich motifs in the human proteome

**DOI:** 10.1186/s12864-025-12132-5

**Published:** 2025-10-06

**Authors:** Eric Schumbera, Dorothee Dormann, Andreas Walther, Miguel A. Andrade-Navarro

**Affiliations:** 1 Institute of Organismic and Molecular Evolution, Faculty of Biology, Johannes Gutenberg University, Hanns-Dieter-Hüsch-Weg 15, Mainz, 55128 Germany; 2 Institute of Molecular Physiology, Johannes Gutenberg University, Hanns-Dieter-Hüsch-Weg 17, Mainz, 55128 Germany; 3 Institute of Molecular Biology (IMB), Ackermannweg 4, Mainz, 55128 Germany; 4 Life-Like Materials and Systems, Johannes Gutenberg University, Duesbergweg 10-14, 55128, Mainz, 55128 Germany

**Keywords:** Arginine–glycine-rich motifs (RG motifs), Intrinsically disordered regions (IDRs), Liquid–liquid phase separation (LLPS), RNA-binding proteins, Protein sequence composition, Disordered protein function, Human proteome, Computational motif analysis, Aromatic residues

## Abstract

Arginine-glycine (RG)-rich motifs are among the most prevalent RNA-binding elements within intrinsically disordered regions (IDRs) of proteins and play crucial roles in RNA metabolism, gene regulation, and the formation of membraneless organelles via liquid phase separation (LLPS). Despite their biological relevance and implication in neurological disorders and cancer, the sequence features and context dependencies that define functional RG motifs remain poorly characterized owing to their disordered nature and sequence variability. In this study, we present a computational framework to dissect the sequence and structural context of RG motifs across the human proteome. By contrasting a functionally defined positive dataset—enriched for RNA-binding and phase-separating proteins—with a negative dataset of RG motif proteins lacking these annotations, we identified distinct compositional and contextual signatures. RG motifs in the functionally defined positive dataset show increased enrichment of phenylalanine, tyrosine, aspartic acid, and asparagine, both within and around the motif, as well as nonrandom spatial relationships with structured RNA-binding domains. Notably, phenylalanine and tyrosine exhibit divergent positional and functional profiles, suggesting distinct mechanistic roles. Our analysis highlights the potential of sequence-based approaches to uncover functional determinants in disordered protein regions and further advances our understanding of the properties of RG motifs, offering a transferable framework for the study of other low-complexity motifs.

**Supplementary Information:**

The online version contains supplementary material available at 10.1186/s12864-025-12132-5.

## Background

Proteins are determined by their sequences, and within their sequences, we differentiate between domains—long stretches of amino acids with secondary and tertiary structures—and unstructured stretches (intrinsically disordered regions, IDRs). IDRs have various lengths and often contain regions with few amino acid types (low-complexity regions, LCRs) and short motifs that can carry posttranslational modifications and participate in interactions with proteins, RNA and DNA [1].

Arginine–glycine-rich motifs—in this work, called RG motifs but also known as RGG boxes [2], RGG/RG repeats [3], or glycine–arginine-rich (GAR) motifs [4]—are the second-most common RNA-binding motif known in humans and are loosely defined by RG and/or RGG repeats interspersed with various amino acids. They have very different lengths and compositions, revealing the layer of complexity in this motif [2, 3, 5, 6].

Functionally, RG motifs were early on associated with the binding of RNA, shown by the RG motif of the heterogeneous nuclear ribonucleoprotein U (hnRNP U) being evolutionarily conserved and required for its RNA-binding ability [2]. Nowadays, it is clear that RG motifs participate in diverse cellular processes, including transcription [7, 8], chromatin remodeling [9, 10], DNA repair [11–13], pre-mRNA splicing [14, 15], RNA transport [16], and translation [17, 18].

Furthermore, RG motifs have gained increasing attention since they have been shown to play a major role in many phase-separating proteins. Liquid‒liquid phase separation (LLPS) is a condensation process in which a homogenous solution of molecules separates into two distinct phases: a dense phase and a dilute phase [19]. It can lead to the organization of RNA and protein molecules within cells into so-called membraneless organelles or biomolecular condensates (sometimes referred to as granules, bodies, foci or phase-separated droplets) [20]. Well-studied membraneless organelles contain RG motif-containing proteins, such as Laf-1, PGL-1 and PGL-3 in P granules of *Caenorhabditis elegans* [21, 22]; nucleolin and fibrillarin in the nucleolus [23–25]; and many RNA-binding proteins, such as FUS, EWS, TAF15, FMRP, G3BP1 and caprin-1, in stress granules [26–32]. Prominent examples clearly show that RG motifs are often necessary for the phase separation propensity of the protein in which they reside, as observed, for example, for the RG motifs of Laf-1, DDX4, CIRBP and FUS [33–36].

Additionally, RG motif proteins have attracted interest because of their relevance in major diseases, such as fragile X mental retardation syndrome [37, 38], amyotrophic lateral sclerosis [39–43], spinal muscular atrophy [44], and Ewing sarcoma [45–47], and many RG motif-containing proteins, such as RD4, BAZ1A, Drosha, ING5 and hnRNPK, are misregulated in cancer, although the role of the RG motif is unclear in most cases. In nucleolin, however, a pseudopeptide targeting the nucleolin RGG/RG motif reduces its cell surface level via internalization, suppressing breast tumor growth and highlighting its oncogenic role [48, 49].

While there is growing interest in RG motif-containing proteins in the scientific community, computational studies are difficult, in part because RGG/RG repeats occur in intrinsically disordered regions (IDRs) that are often composed largely of limited amino acid types and generally do not contain any stable secondary or tertiary structure [50].

For some proteins, such as hnRNPU, the RG motif is the only identified RNA binding domain (RBD) [2]; this motif is most often found in proteins with other structured RBDs [3, 51, 52]. This relationship between the disordered RG motif and structured, classic RBDs, such as the RNA recognition motif (RRM) and KH domains and zinc fingers, is intriguing but not very well understood.

In this work, we identify sequence features and associated functions of RG motifs in human proteins through computational methods that analyze their sequence and functional context. The application of this approach was necessitated due to the unstructured and poorly conserved nature of this sequence feature, which precludes the application of sequence homology and structural predictions. We present an adapted approach that leverages reported evidence on RG-rich proteins as a means to find significant differences between RG motifs and associate them with distinct functionalities. Our results serve as a foundation for specific experimental research by showing biases in sequence properties and context that could influence RG motif behavior and function.

## Methods

### Data acquisition

The one-sequence-per-gene version of the reference human proteome (”UP000005640_9606.fasta”) was downloaded from the Universal Protein Knowledgebase—UniProt— [53] in January 2023. Within this proteome, the proteins were annotated with Gene Ontology (GO) terms via the QuickGO API [54]. The phase separation propensity for the human proteome was collected from PhaSePred, a meta-predictor for phase-separating proteins [55], and the feature “*SaPS-8fea*” with a cutoff of 0.5 was used to define the proteins into phase-separating proteins (*SaPS-8fea* > 0.5) and non-phase-separating proteins (*SaPS-8fea* <= 0.5). Annotation of intrinsically disordered regions (IDRs) in the human proteome was performed through the API of MobiDB [56]. The metric used for determining whether a region is disordered or not is ‘*prediction-disorder-mobidb_lite*’, which is a consensus prediction value, where at least 5/8 of the predictors must agree on the disorder prediction to assign either a disordered state or a structural state at the residue level. Domains inside the proteins were annotated through the InterPro API, hosted by EBI [57]. Although InterPro provides annotations from various sources (smart, Pfam, CDD, and prosite), only domain annotations from Pfam were taken into consideration in this work to simplify the domain annotations. Annotations for posttranslational modification sites were acquired through the UniProt API hosted by EBI [58].

Via the glycine-arginine-rich (GAR) motif finder tool, the entire human proteome was analyzed for RG motifs [4]. The definition for RG motifs provided in that work is also used consistently in this work. Further filtering consisted of scrapping discovered RG motifs if they were fully in non-disordered regions, since we expect (functional) RG motifs to exist only in disordered regions. Finally, all collagen-related proteins were removed because the collagen sequence pattern was selected by the GAR motif finder, which was used to define the RG motif in this work.

### Dataset preparation

To perform statistical analyses between two groups, a positive (“functional”) and a negative (“nonfunctional”) protein set were needed. To achieve this goal, all human proteins with a predicted phase separation propensity (PhaSePred), all human proteins with at least one annotated nucleic-acid (NA)-binding-related (including child terms) GO term (QuickGO) and all human proteins with at least one RG motif in their sequence (GAR-motif finder, [4]) were overlapped in a Venn diagram to create 7 distinct subsets .

The positive (“functional”) set was defined as the group of proteins that contained at least one RG motif and were both predicted to phase separate and annotated with at least one NA-binding-related GO term, the center subset of the Venn diagram. Consequently, the negative (“nonfunctional”) set was defined as the group of proteins that contain at least one RG motif but are neither predicted to phase separate nor annotated with at least one NA-binding-related GO term, the bottom subset in the Venn diagram. This resulted in datasets of 193 and 230 proteins, respectively.

The list of GO terms classified as child terms for NA binding was gathered through QuickGO and can be found in supplementary material S1. The list of proteins containing RG motifs and their functional annotations (phase separation characteristics and NA-binding annotations) can be found in supplementary material S2.

### Statistical analysis

All the statistical tests between the positive and negative sets were conducted as Mann‒Whitney significance tests, and Benjamini‒Hochberg correction was applied, if applicable. The results were annotated as follows: “ns”: *p* value > 0.5, “*”: 0.5 > *p* value > 0.1, “**”: 0.1 > *p* value > 0.01, “***”: 0.01 > *p* value > 0.001, “****”: *p* value < 0.001. Where possible, the log2-fold change of means was used to quantify the change between the datasets; otherwise, if the means were close or even exactly 0 (and thus making log2-fold change an ineffective method), Cohen’s d value was used to measure the effect size by dividing the difference between the means of the datasets by the pooled standard deviation.

Distances between the RG motif and an annotated domain were calculated from the center of the motif to the center of the domain. For the analysis of sequence properties around the motif, blocks of ten residues left or right of the motif were analyzed; however, only blocks that were still fully within an IDR were considered.

Considering that the positive and negative datasets contain a relatively low number of sequences (193 and 230 proteins, respectively), when calculating amino acid proportions by position in these datasets, a sliding window method was applied. A window of 4 residues away from the motif was considered for the calculation of the amino acid composition, and then the proportion of all amino acids was calculated across the windows for all proteins of the respective subset. This leads to smoother curves and better visualizes certain trends, while the payoff is that potential localized signals are lost. Additionally, similar to before, a window is considered for analysis only if it is still fully located within an IDR. Additionally, for comparison, we calculated the average amino acid composition in all human IDRs and added it to the plots as a dotted line.

### Programming packages and code availability

All the research was performed with popular python packages, such as *pandas*, *matplotlib*, *seaborn*, *scipy*, *gseapy* and *biopython*. The *localCIDER* package [59] was used to calculate the fraction of disorder-promoting residues, net charge per residue (NCPR) and hydrophobicity. The jupyter notebooks that were built for data acquisition and the statistical analyses as well as the visualization and intermediate processed files can be accessed via a github repository (https://github.com/erschumb/hu-RG-motif-composition-analysis).

## Results

### Human RG motif statistical comparisons reveal significant differences in physicochemical properties

**Fig. 1:**
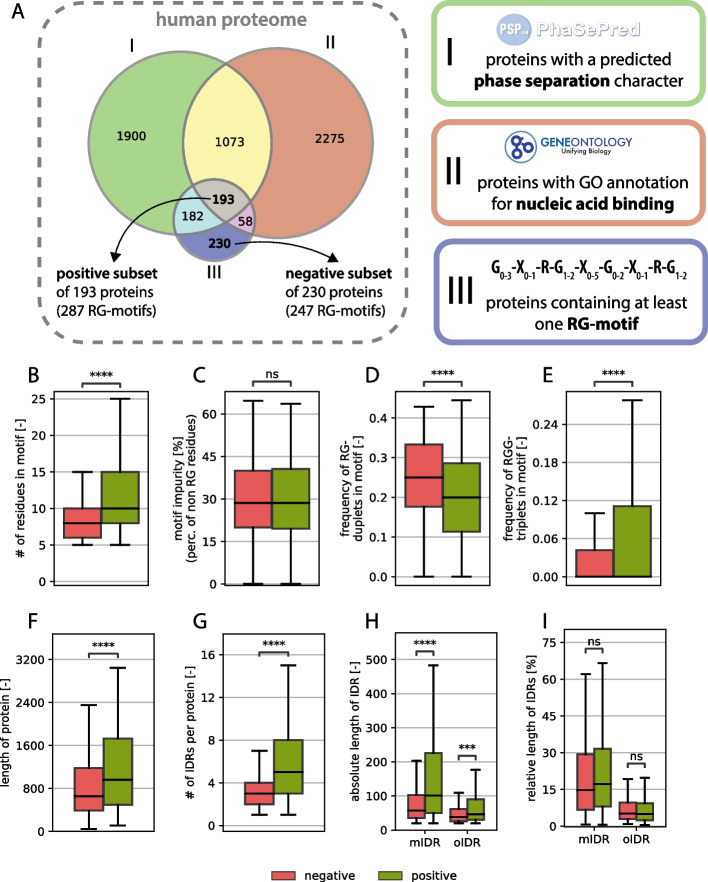
**A** Venn diagram of protein sets with predicted phase separation characteristics, nucleic acid binding and RG motif-containing proteins, which defines the positive and negative subsets. **B**-**I** General comparative analysis between RG proteins from the positive set (green) and the negative set (red) mIDR (motif-containing IDR): IDR in which the RG motif is located, oIDR (other IDR): IDR without any RG motif.

To understand important characteristics or properties of an RG motif, their properties were compared between a positive (“functional”) set and a negative (“nonfunctional”) set. These two groups were defined by classifying human proteins containing at least one RG motif (pattern definition shown in Fig. 1A) into positive and negative sets on the basis of phase separation prediction and NA-binding annotation. The positive set (193 proteins) included those predicted to phase separate and annotated with at least one NA-binding GO term, whereas the negative set (230 proteins) lacked both features (see Methods for details).

While the average length of the RG motifs seems to be greater in the positive set (see Fig. 1B), the percentage of amino acids, which are neither arginine nor glycine (here called impurity), does not differ between the groups (see Fig. 1C). This underlines the problem of defining a clear RG motif, since impurities do not affect the function as much as they do for structured regions.

The number of RG duplets vs. the number of RGG triplets reveals an opposing image, where the number of RGG triplets is greater in the positive group, whereas more RG duplets can be found in the nonfunctional group (Fig. 1D, E). Notably, only the RG duplets that do not have a glycine residue in the following position are counted because that would create an overlap between the RG duplet count and the RGG triplet count. Although there are well-studied RG motifs consisting largely of RG duplets (314 isoforms found with a tri-RG motif [3]), they seem to occur less in the positive dataset than in the negative dataset.

Compared with those in the negative dataset, the proteins in the positive dataset were significantly longer (Fig. 1F), suggesting that functional RG motifs are preferentially found in larger proteins. While this relationship has not been systematically reported, our results indicate a novel link between RG motif functionality and host protein length. Furthermore, the number of intrinsically disordered regions (IDRs) per protein was significantly greater in the positive dataset (Fig. 1G), reinforcing the established link between RG motifs and disordered protein domains. IDRs provide structural flexibility, facilitating transient interactions with nucleic acids and other biomolecules, which is a hallmark of RG-containing RNA-binding proteins [60]. This, of course, is directly related to host protein length; however, it has never been directly shown.

The absolute length of IDRs was also significantly greater in proteins from the positive dataset (Fig. 1H). When distinguishing between motif-containing IDRs (mIDRs)—disordered regions that contain at least one RG motif—and other IDRs (oIDRs)—disordered regions that lack an RG motif, mIDRs are significantly longer in the positive dataset than in the negative dataset, suggesting that functional RG motifs tend to appear within extended disordered regions. This association is however correlative, since there is no sign that longer mIDRs cause increased motif functionality. For oIDRs, we also observed a length increase, but this increase was not as strong (Fig. 1H). Interestingly, the relative length of IDRs (expressed as a percentage of total protein length) did not differ significantly between the two datasets, indicating that while RG-containing proteins tend to have longer IDRs, the proportion of disordered content within a given protein remains consistent. This can also be observed for the oIDRs. The effect that remains consistent across an absolute or relative perspective is that mIDRs are generally much longer than oIDRs are, regardless of the set. These observations support the hypothesis that RG motifs are functionally linked to protein disorder and are preferentially embedded within longer IDRs, where they may contribute to RNA-binding and phase-separating functions [61]. However, we notice that the negative subset also has longer mIDRs than oIDRs so the embedding of RG motifs in longer IDR stretches seems to be independent of their functionality but clearly not independent to the existence of an RG-motif. Furthermore, no significance in the positioning of the RG motif within the IDR was found, suggesting that there is no generally necessary position of the motif within the IDR (see supplementary material S3). This result is consistent with a general lack of positional bias found for all types of compositionally biased regions within the IDRs of human proteins [62].

Taken together, the general findings resulting from the comparison of the RG motifs in the positive and negative datasets confirm many previous independent observations about RG motif functionality. Beyond this, we aimed to underline the validity of the separation of the RG-rich proteins into positive and negative sets, by comparing the number of methylations in and around the RG-motifs between the two sets. We observed that there was a significant enrichment of Omega-N-methylarginine and asymmetric dimethylarginine in the positive set (*p*-values 2.4e-08 and 2.0e-07 respectively, Mann–Whitney U test), while other methylation types do not appear at all in the negative set. Since arginine methylation is a well-known regulation mechanism in RG-motifs, we conclude that the approach presented here is valid.

### RG motifs appear along a large variety of domains and maintain specific distances from them

RG motifs can act as functional elements on their own within a protein, but they can also enable, fine-tune and enhance the function of structural domains in a protein. For example, the RG motif in Scd6 (the yeast homolog of LSM14A) is solely responsible for translational repression [63], whereas the RG motifs in FMRP, hnRNPU or FUS increase binding to RNA and fine-tune the RNA-binding specificity of those proteins [6, 64].

**Fig. 2:**
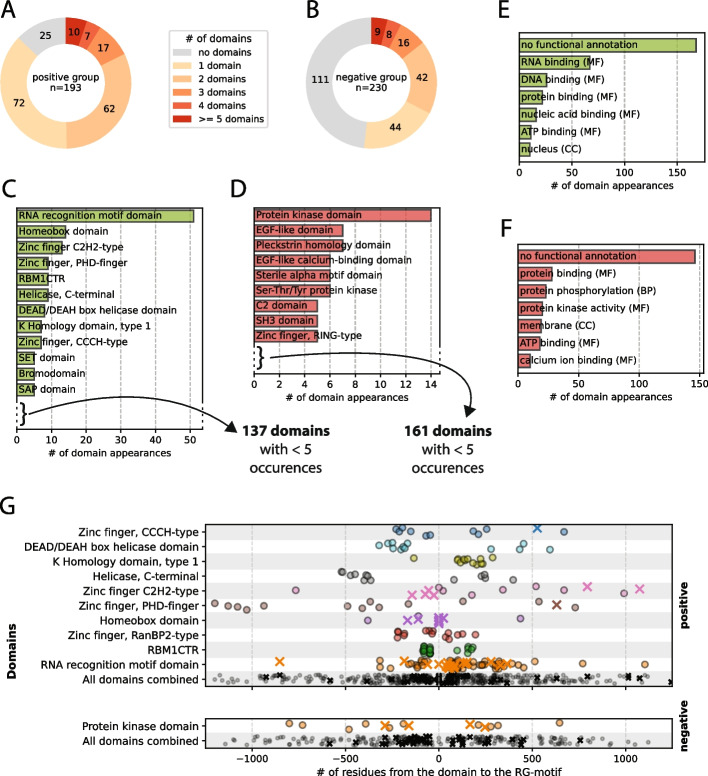
**A**-**B** Distribution of the number of domains per protein for the positive and negative sets. **C**-**D** Domain types sorted by the number of occurrences in the positive and negative sets, respectively. Domains with fewer than 5 occurrences are not shown. **E**–**F** Top 10 most common GO term types for the annotated domains of the positive and negative sets, respectively. **G** Distributions of the distances between an RG motif and annotated domains that appear together at least 10 times. A cross indicates a case where a protein has exactly 1 domain and 1 RG motif annotated.

In the positive set of proteins, we observed 25 proteins (out of 193) without any domain annotated to it, suggesting that the RG motif could be the only RNA binding element and solely responsible for the nucleic acid binding character (see Fig. 2A). Most RG motifs co-occur with domains, indicating that the RG motif often functions in combination with structured domains to enable, enhance or fine-tune certain functions. The finding that a much larger share of proteins in the negative set did not have any annotated domains in the protein (111 proteins out of 230) suggests that functional RG motifs functioning on their own are most likely less frequent and rare (see Fig. 2B).

Owing to the functional filtering of the positive dataset (see Sect. 2.2), some very well-studied RNA/DNA-binding domains, such as the RNA recognition motif (RRM), homeobox domains and a subset of the zinc-finger domain family, appear in the positive set of the human RG proteome, whereas they are missing in the negative set (see Fig. 2C, D). However, the amount of domain variety that appears is surprising, since in 168 proteins in the positive dataset with at least one domain, we find 149 distinct domains appearing 330 times in total. Over 50% (168 out of 330) of the domain appearances in the positive dataset are not annotated with any GO term, which underlines the missing knowledge about functionality in the RG proteome (see Fig. 2E). However, the case is even more extreme in the negative dataset—170 different domains appearing 258 times, with almost 60% (146 out of 258) being of unknown function—which might be due to many annotated domains not being properly understood in their function (see Fig. 2F). Understanding the function of the domains might help understand exactly how the RG motif – provided it is a functional motif – works collectively with the domain.

Since an RG motif—by our definition (see Methods)—exists in a disordered region, an RG motif that works collectively with a domain does not have to be directly adjacent to the domain but could theoretically, owing to the flexibility of the disordered region, be located apart from the domain. Many well-studied RG proteins have relatively large sequence distances between the domains and the RG motif. FUS has an RG motif that is 40 residues away from the zinc-finger domain and over 100 residues apart from another RG motif. The heterogeneous nuclear ribonucleoprotein A1 (hnRNPA1) motif contains an RG motif that is 40 residues apart from the first RRM and more than 100 residues apart from the second RRM.

When comparing distances between domains and RG motifs, we observed the distance values clustering together specific to their domain types (see Fig. 2G). While no clusters or patterns emerge when comparing the entirety of domain‒motif distances across the datasets, they can be detected when looking at the domains individually, suggesting that the distance is related to the specific functionality of the domain.

While some domains show clear singular clusters (e.g., K homology domain (type 1), Sam68 (tyrosine-rich) domain or KHDRBS (Qua1) domain), others show a more homogeneous distribution (e.g., the zinc-finger types), but with visible differences in the overall proximity to the motif. Additionally, some domains appear (almost) exclusively in either the N- or C-terminus of the motif, whereas others seem symmetrically distributed between the N- or C-termini. A clustering of distances across unrelated proteins suggests that there is either a functional connection between the domain and the motif and/or an evolutionary reason for the consistency. If that is the case, a “preference” of a side (N-terminal or C-terminal) suggests that there is some mechanistic association between the two elements.

An analysis of the domains grouped by annotated function (GO terms) did not reveal any patterns or clusters. This finding indicates that the positioning of the RG motif relative to a domain is not correlated with the overall function but rather, more specifically, with the specific mechanism of a certain domain type. The residues that separate the RG motif and the domain type could act as linker elements and, depending on the functional mechanism, are optimized to reach a certain length.

This domain analysis in the context of RG motifs shows that distances between RG motifs and domains could be a useful property to determine whether an RG motif interacts functionally with a domain.

### Amino acid composition analysis reveals biases and properties of “true” RG motifs

Composition analysis of disordered regions has been a difficult area of research because of the naturally high variance in sequences within these regions. Here, we attempt a systematic approach by comparing the amino acid compositions of the positive and negative protein sets to identify biases or trends, despite the high variance of IDRs.

**Fig. 3:**
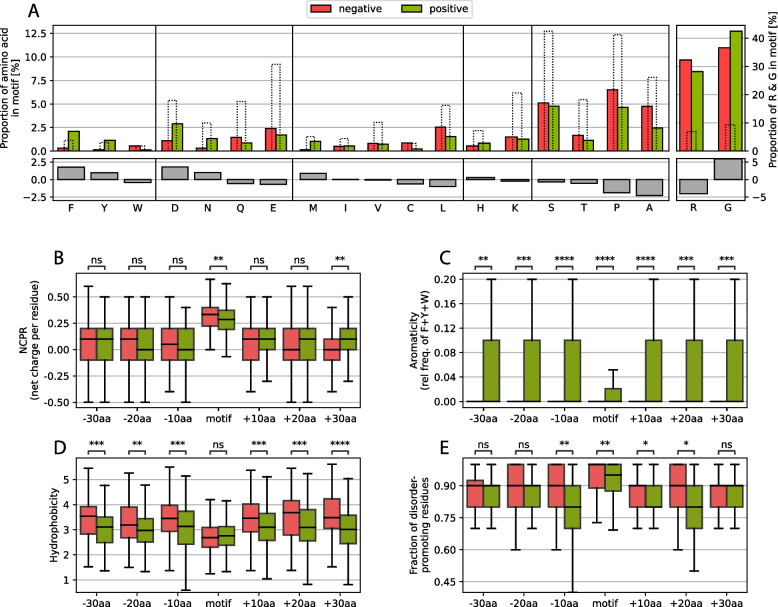
**A** Changes in amino acid frequency between the positive and negative sets of motifs, grouped by amino acid type. The dashed bars indicate the average amino acid proportions calculated for all human IDRs. **B**-**E** Comparative analysis of the NCPR, aromaticity, hydrophobicity and disorder-promoting residue fraction of the motif and the regions of 10 residues, N-(left) and C-terminal (right) of the motif.

First, we compared the amino acid composition of the RG motif of both datasets and revealed that phenylalanine (F), aspartic acid (D), and asparagine (N) increased by more than 1% in the positive set compared with the negative set, whereas tyrosine (Y) and methionine (M) increased slightly less than 1% (see Fig. 3A). Notable decreases of more than 1% are only observed for alanine (A) and proline (P), with leucine (L) barely missing the 1% mark. As expected, arginine and glycine are more common than average in all human IDRs, but this is also the case for tyrosine and phenylalanine. These results are not consistent with classical amino acid groupings (aromatics, positively charged, negatively charged, uncharged and hydrophobic), especially since tryptophane (W, as the third aromatic amino acid) is slightly decreased, whereas glutamic acid (E, as the second negatively charged amino acid) and histidine (H) and lysine (K) (the two other positively charged amino acids) barely change. Tyrosine and phenylalanine have previously been associated with RG motifs [4, 65].

Arginine and glycine content differences are also shown in the graph, and while the arginine content is lower, the glycine content is higher in the positive set, which is consistent with the finding that RGG triplets are more prevalent in the positive set than are RG duplets.

In general, these results raise the question of the extent to which the chemical properties of charge, aromaticity and hydrophobicity play a role in affecting the functionality of the RG motifs and whether other (usually more complex) properties, such as the propensity to form secondary structures, or the chemical groups of the amino acid side chains (amino groups, guanidium groups, etc.) are more important for their role in RG motifs.

To answer these questions, we compared the amino acid properties (net charge per residue (NCPR), aromaticity, hydrophobicity and fraction of disorder-promoting residues) in and around the motif. Here, we observed no significant differences in NCPR directly around the motif; however, only in the motif itself was the NCPR slightly greater in the negative dataset, possibly because of the greater percentage of arginine (Fig. 3B). This finding strongly suggests that the charge itself is most likely not the main driving force behind a functioning RG motif or at least not enough to explain their full function, which has been indicated in past works by observing loss of function through mutation of arginines to lysines [35].

The aromaticity shows a very strong and uniform signal of being more prevalent in the positive set, which is underlined by the findings concerning tyrosine and phenylalanine mentioned above (Fig. 3C). The importance of aromaticity (not including tryptophan, which was found more often in the negative set; Fig. 3A) is well known for RG motifs, but it seems that the presence of aromatic compounds extends far beyond the motif itself.

Despite the differences in the frequency of aromatic residues, the hydrophobicity of the adjacent regions is reduced in the positive set, suggesting that a large increase in hydrophobicity directly around the motif might affect how well the RG motif is available to its hydrophilic environment (Fig. 3D). Strong hydrophobic forces in sequences usually appear inside folded domains, facilitating the folding and exposition of hydrophilic residues to the surface. If strong hydrophobic forces surround the motif, the RG motif could be hidden by hydrophobic residues clustering together around the RG motif, thus making it difficult for the motif to be accessed by interacting partners or domains, which would effectively limit their functionality and explain the prevalence in the negative dataset.

Therefore, we also analyzed the fraction of disorder-promoting residues (Threonine, Alanine, Glycine, Arginine, Aspartic Acid, Histidine, Glutamine, Lysine, Serine, Glutamic Acid and Proline are considered disorder-promoting according to [59]). Inside and closely around the motif, a lower fraction of disorder-promoting residues can be found in the positive set (Fig. 3E). This could suggest that possible secondary structures might arise under certain conditions. Since RG motifs are regions of low complexity, no general structure has yet been defined for RG motifs. However, in the RG motif of nucleolin (NCL), which is rich in RGGF repeats, repeated β-turns are the major structural component that is observed [66]. In fragile X mental retardation protein (FMRP), arginines at positions 533 and 538 have been shown to form intramolecular contacts with the RNA duplex-quadruplex junction [67]. Notably, the residue at position 532, which is directly adjacent to the first arginine, is a phenylalanine. Additionally, RNA-binding protein EWS (EWSR1) is associated with G-quartets and contains many phenylalanine residues within its RG motif [68]. Thus, we find evidence of substructures in RG motifs, which should be evaluated more closely.

**Fig. 4:**
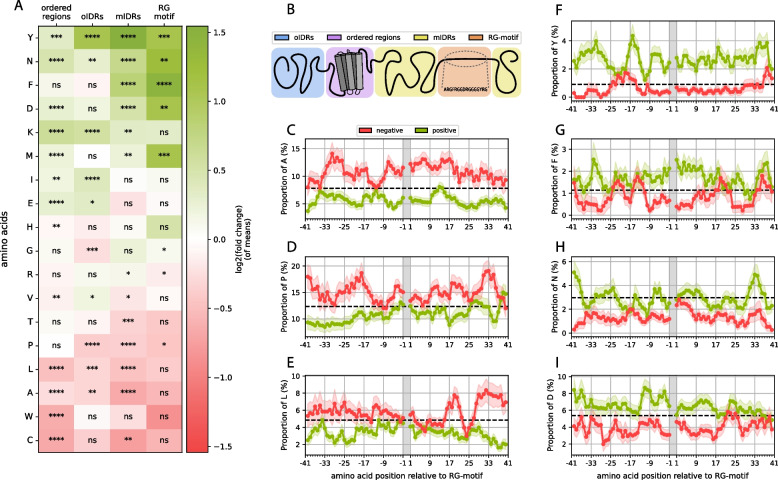
**A** Positional enrichment heatmap of the amino acid composition of the positive vs. negative subsets (positive log. fold change (green): enrichment in pos. dataset; negative log. fold change (red): depletion in neg. dataset) when separated into 4 regions, as shown schematically in (**B**). **C**-**I** Amino acid compositions at the residue level of the regions left and right of the motif acquired through a sliding window approach with error bars as transparent background areas and the average proportion of each amino acid calculated for all human IDRs as dotted lines.

To expand the analysis, we also examined the amino acid composition of the entire protein, which was separated into regions with different structural propensities and relationships with the RG motif (Fig. 4A, B). We differentiated between 4 regions: the actual RG motif (1), the motif-containing IDR (mIDR), (2), other IDRs (oIDRs) in the protein (3) and structured regions (4). Tyrosine (Y) and asparagine (N) seem to be enriched over the entire protein, which could be associated with their functions, for which they were selected. The protein composition depends on the context, including the subcellular location [69]. Phenylalanine (F) is enriched only in the direct vicinity of the RG motif or in the motif itself, therefore showing a different image than tyrosine (Y). Aspartic acid (D) stands out, especially since its negative charge can inhibit or promote phase separation depending on the sequence context, particularly in relation to arginine-rich motifs, as well as the overall charge patterning of intrinsically disordered regions [65, 70, 71]. Also visible is the enrichment of lysine everywhere except in the RG motif itself. Lysine has been shown to have a weaker phase separation propensity and is outcompeted by arginine for negatively charged partners [72]. Furthermore, acetylated lysine can even reverse lysine-driven phase separation [73, 74], suggesting that lysine should not appear within RG motifs, which is also visible in the figure. However, the enrichment of lysines outside of the RG motif in both ordered and disordered parts of the protein suggests a possible further role of this motif in terms of RG-rich proteins. Finally, we note the enrichment of methionine, which has been associated with a role in regulating LLPS [75, 76].

Significant amino acid depletion does not occur directly in the motif; however, the observed fold changes indicate depletion of a group of hydrophobic residues (most notably tryptophan) but is not statistically significant, most likely due to the small sample size. Cysteine, leucine, alanine, threonine and proline are significantly depleted in mIDRs, which fits previous observations, especially the lower hydrophobicity around the motif in the positive set.

Some other signals could lead to interesting yet unknown insights into the RG motif context. For example, we do notice an enrichment of isoleucine (I) in oIDRs. Additionally, as mentioned above, lysine was enriched throughout the entire protein except for the RG motif itself. Similarly, glutamic acid is enriched in the structured parts of the protein. The protein may balance out functionally similar amino acids (such as D and E, R and K or I and L), and since aspartic acid and arginine are used heavily in and around the motif, glutamic acid or lysine are used more frequently in other areas of the protein to correct the overall imbalance of usage in other parts of the protein. There are studies that show that there is selection pressure in organisms not only to use low-cost amino acids but also to balance out the usage of certain amino acids, since the availability of heavily used amino acids will be lower and, therefore, the synthesis of the protein overall will be lower [77]. However, it is also possible that the increase in these amino acids could imply functional aspects.

For a more detailed composition analysis, we specifically looked at the amino acid compositions around the motif at the residue level (Fig. 4C-I). We applied a sliding window method (see Methods; Chapter 2.3). These results show that the two aromatic residues (F and Y), which we previously identified as strong signals, manifest very different profiles. While phenylalanine is strongly enriched (fold change of 1.5) in the motif and less enriched but still significantly enriched in the mIDR, the phenylalanine signal outside the motif ends after 10 residues left or right of the motif. Tyrosine, however, seems to be even more enriched outside the motif but weaker in the RG motif itself. This is surprising considering the similar chemical properties of the two amino acids and given that both have been strongly associated with RG motifs and are necessary for function [65, 78, 79].

Another interesting fact we observed is that in the case of asparagine (Fig. 4H), the average proportion shown is comparable to the human IDR average, but the average proportion in the negative dataset is far below the human IDR average. This is in contrast with tyrosine, for example, where the average proportion in the negative set is close to the human IDR average of tyrosine, and the average proportion is much greater (Fig. 4F). Aspartic acid and phenylalanine show mixed images (Fig. 4I and G, respectively). It is unclear what this result could imply at this stage, however, this could provide an interesting and fruitful hypothesis to test.

Additionally, the frequency profile of leucine seems intriguing since the composition of the positive and negative sets is not different directly adjacent to the motif but starts diverging only after approximately 20 residues outside the motif (Fig. 4E). This could reflect constraints on the composition of the sequences surrounding the RG motif associated with the physicochemical properties of the motif independent of function.

Notably, the arginine and glycine proportions are strongly above the human IDR average for both the positive and the negative sets, even outside of the actual motif, particularly for arginine (see supplementary material S4). This strongly suggests that the actual RG motif might extend far beyond the definition used in this work and previous definitions. It is more likely that a whole region must be considered for its functionality, and it would be vital to determine if and where the functional cutoff in terms of arginine and glycine content would be.

In the following chapter, we conducted deeper computational analysis on one of the clearest signals, which are the phenylalanine and tyrosine enrichment profiles.

### Opposing tyrosine and phenylalanine profiles in RG motifs suggest different roles of the two aromatic residues

The clearest signals that could be observed in the amino acid composition analysis were the signals of F/Y and how they affected the entire aromaticity of the motif and the surrounding region (up to 30 residues in the N- and C-termini of the motif) to be enriched in the positive dataset. The role of aromaticity in the RG motif is well known; however, the difference between these two aromatic compounds, in addition to the possible phosphorylation of tyrosine, is unclear. Thus, we applied further computational research.

**Fig. 5:**
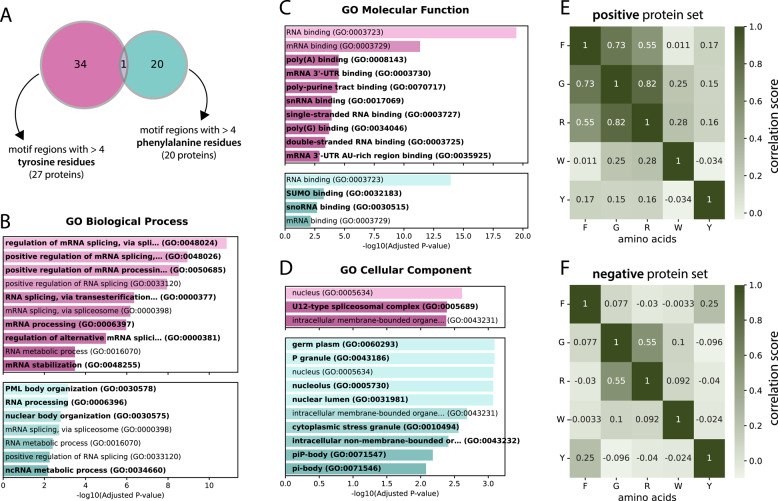
**A** Venn diagram of groups with at least 5 tyrosine (pink) and at least 5 phenylalanine residues (blue) in their motif region. A motif region is defined as the motif itself and the 30 residues left and right of the motif. **B**-**D** Enrichment analysis of the 2 protein sets from (**A**) in terms of GO biological processes, molecular functions and cellular components. The labels that are unique to either the tyrosine or phenylalanine set are marked in bold. **E**- **F** Correlation matrices for selected amino acids for the motifs of the positive and negative sets, respectively

To understand the relationship between phenylalanine and tyrosine frequencies, we overlapped all proteins containing at least 5 tyrosines/phenylalanines in the RG motifs or the surrounding regions of 30 N- and C-terminal residues (analogous to the analysis in Fig. 4C–I) to determine whether there was any overlap between the proteins with “phenylalanine-rich” and “tyrosine-rich” motif regions (see Fig. 5A). The overlap is minimal, and only one motif region contains at least 5 residues of both tyrosine and phenylalanine. This small overlap created the opportunity to perform an enrichment analysis to find possible different functions for the distinct sets of proteins containing either of these regions.

Indeed, we detected differences in the biological processes, molecular functions and cellular components of the two protein sets via GO term enrichment analysis (Fig. 5B, C and D, respectively). While the tyrosine set seems to show a much stronger connection to spliceosome-related processes (four unique spliceosome-related processes and generally lower *p* values) and is associated with the cellular component “U12-type spliceosome complex”, the phenylalanine set is rather involved in nuclear body organization, with PML body organization actually being the most significant biological process visible, as are cellular components such as P granule (and its subcomponents piP-body and pi-body), stress granules and the nucleolus. This clear distinction could suggest unique functions of phenylalanine-rich motif regions versus tyrosine-rich motif regions and provides a strong case for further experimentation.

To further support the notion of a potential regulatory role of tyrosines within or adjacent to RG motifs, we examined the occurrence of phosphotyrosine sites in the positive and negative sets. In the negative set (230 proteins), only four phosphotyrosine sites were detected within mIDRs, whereas the positive set (193 proteins) contained 21 such sites. This represents a more than five-fold increase in the positive set, suggesting that tyrosines in the vicinity of RG motifs are preferentially phosphorylated and may contribute to regulatory functions. Importantly, this effect persists even after normalizing for the total length of mIDRs, which is higher in the positive set (37,157 residues) compared to the negative set (19,556 residues), although the enrichment is somewhat reduced.

Furthermore, we compared the Pearson correlation coefficient between the occurrences of the amino acids within the RG motifs. In addition to an obvious and expected correlation between arginine and glycine (0.82 in the positive set and 0.55 in the negative set), the only amino acid with which we observed any correlation was phenylalanine, with both glycine and arginine values of 0.73 and 0.55, respectively (Fig. 5E–F; full correlation matrices in supplementary material S5). These findings suggest that phenylalanine can play a role in RG motifs, possibly by emerging as a pattern together with arginine and glycine. One pattern that has already been mentioned and appears in well-known RG motif-containing proteins, such as FUS, EWS and TAF15 (FET family), is the RGGF tetramer. Since tyrosine shows no correlation, this result again underlines that it manifests in a different way than phenylalanine, but is still very prevalent in RG motifs and surrounding regions.

## Discussion

RG-rich regions are frequent within IDRs and are associated with functions in RNA and DNA binding as well as in driving LLPS. Some mechanistic evidence of the function of RG-rich regions exists for particular cases. However, a general characterization of RG-rich regions and their functions is still lacking. The variability of IDR sequences and their lack of structure require different approaches compared with those of globular domains, which rely on sequence homology and structural predictions. Sequence analyses of proteins with RG motifs have led to promising motif definitions: the RGG box [2], RGG/RG repeats [3], and glycine-arginine rich (GAR) motif [4].

Following the hypothesis that RG-rich regions with functions in RNA and DNA binding and in LLPS are involved in driving these functions and that RG-rich regions could have other functions or no function at all, here, we used functional and sequence context to segregate proteins with RG motifs into two groups of predicted or known functions: a positive one involved in nucleic acid binding and LLPS and a negative one involved in neither of those functions. For LLPS we rely on one of the latest LLPS predictors at the time, since we would not be able to perform this systematic investigation with purely experimental data since the data is still too scarce. This of course implies that predicted LLPS propensity does not necessarily reflect functional contributions of RG motifs themselves. Nevertheless, we used these predictions only as a broad categorization tool to investigate general sequence trends, and we interpreted the results with caution. Future methodological advances and larger experimental datasets will be important to validate and refine these findings. Also, any biases introduced by the predictors, such as sequence features, on which the predictor is trained, do not influence the results of our analysis, since any biases cancel each other out, since both the positive and the negative set are equally affected.

Furthermore, we decided not to use additional functional features of RGG/RG motifs, such as arginine methylation by PRMTs or binding to Tudor domains. While these properties are highly characteristic of RGG/RG motifs, our aim was to focus on a minimal set of broadly predictable features to maintain statistical power. Future studies could refine the positive set, for example by incorporating interaction data with Tudor-domain proteins, to further validate and extend our findings. However, we did use an analysis of arginine methylation types to confirm that the separation of the dataset into positive/functional and negative/non-functional was sensible, by showing that the positive set contains a strong enrichment of arginine methylation sites, which is very characteristic of functional RG-motif. This strongly supports the validity of our approach.

These protein datasets allowed us to identify sequence features and protein functions at different levels of RG motif-containing proteins. The reduced frequency of hydrophobic residues surrounding the RG-rich region in the positive dataset suggests general mechanisms that increase the accessibility of the motif and its interactions (inter- or intramolecular). Clusters of motifs accumulate at fixed distances from some domains, sometimes specifically at one side of the domain, which suggests some degree of structural interaction, which needs to be studied further for mechanistic insights. The increased frequency of the aromatic amino acids tyrosine and phenylalanine within the RG motif and its vicinity suggests that these residues play a role in the function of RG motifs, which has been experimentally observed and discussed for particular proteins but has never been systematically characterized. While the frequency of both of these residues around RG motifs is higher in the positive set than in the negative dataset, the way they appear is very different, with tyrosine having a homogeneous increase in frequency around the motif in a wide region (> 40 amino acids), whereas the phenylalanine frequency is stronger in the motif itself and only appears directly around the motif (< 10 amino acids). These differences manifest at the functional level, with tyrosine-rich RG motifs associated with splicing and phenylalanine-rich RG motifs associated with nuclear body organization. This finding provides great support for better understanding the role of the amino acids in the RG motifs, can be extended to other signals found in this study and provides novel targets for functional assays.

Our findings also underscore that the current definition of RG motifs is incomplete and warrants revision. The presence of proteins such as FUS, which contains both RGG triplets (18 instances) and RG duplets (4 instances), suggests that these forms may function together and should not be considered separately. This challenges classifications on the basis solely of RG/RGG repeats, such as RGG boxes (di-/tri-RG/RGG repeats) [3]. Moreover, even motif-based definitions such as the RG pattern used in this study [4] appear insufficient. We observed that residues such as tyrosine, phenylalanine, asparagine, and aspartic acid are enriched not only within but also up to 40 residues outside the motif. Surprisingly, this extended enrichment pattern also included arginine and glycine. These findings suggest that classical RG motifs may represent only the core of a broader yet undefined, compositional or functional motif.

Furthermore, RG motifs co-occur with a diverse array of domain types and show domain-type–specific distance patterns, suggesting potential functional relationships. We also identified a small group of RG-rich proteins that function without the need for a structured domain. These RG motifs may represent a distinct subclass and warrant further investigation to understand how RG motifs can mediate function in the absence of nearby domains. Comparing these with domain-associated RG motifs could reveal mechanistic differences in their mode of action.

Importantly, the positive dataset in this study was constructed via a phase separation predictor, primarily to increase the dataset size. This was necessary because experimental, proteome-wide annotations of phase separation remain limited, which would otherwise restrict the statistical power of our analyses. Similarly, although the inclusion criterion of a GO term related to nucleic acid binding was used to gather relevant proteins, its presence does not imply that RG motifs are directly responsible for this function. The observed activity may instead come from structured domains or other sequence features. Nonetheless, the consistency of our findings with recent studies on RG-rich proteins supports the validity of our approach. By combining predictive tools with rigorous sequence analysis, this method enables the identification of compositional patterns and functional associations in highly variable disordered regions that are often missed by domain-centric or motif-only analyses. As such, it represents a powerful and scalable strategy for uncovering subtle but biologically meaningful sequence signals in disordered proteomes.

A potential limitation of our analysis is the inclusion of paralogous proteins, which in principle introduced redundancy. However, only 46% of proteins in the positive set and 13% in the negative set belong to paralogous groups, and over two-thirds of these paralog pairs share less than 50% full-length sequence similarity. Because intrinsically disordered regions typically diverge even more rapidly than structured domains, the effective overlap among paralogs is likely lower still in the regions we are studying. Excluding paralogs would require arbitrary decisions about which representatives to retain and would result in a smaller dataset, which in turn would lead to a loss of information about proteins with RG-rich motifs, so we opted to include all paralogous proteins while acknowledging this as a minor source of potential redundancy.

## Conclusion

In summary, our systematic composition analysis provides a novel framework for identifying substantiated composition analysis properties of RG motifs for further experimental verification or deeper computational analysis. We confirm existing knowledge about the amino acid composition of RG motifs and add novel insights, particularly regarding the role of the aromatic residues tyrosine and phenylalanine. Our work opens the door to adapting this analysis framework to other motifs and regions in disordered sequences, where traditional methods from structural biology struggle, and further the understanding of the sequence context of RG motifs.

## Supplementary Information

Supplementary Material 1

Supplementary Material 2

Supplementary Material 3

Supplementary Material 4

Supplementary Material 5

Supplementary Material 6

## Data Availability

All data and code supporting the findings of this study are available in the following GitHub repository: (https:/github.com/erschumb/hu-RG-motif-composition-analysis). This includes the final figures, intermediate data files, and most raw input files (except those retrieved via external APIs, which are documented in the repository's README).

## Abbreviations

RG-rich: Arginine/Glycine-rich
LLPS: Liquid-liquid phase separation
IDR: Intrinsically Disordered Region
GAR: Glycine-Arginine rich
mIDR: Motif-containing IDR
oIDR: Other IDR
RRM: RNA recognition motif
NA-binding: Nucleic-acid-binding
RBD: RNA-binding domain
LCR: Low complexity region
NCPR: Net charge per residue
