# Supplementary figures and images for: Computational investigation of the sequence context of arginine/glycine-rich motifs in the human proteome
Source: BMC Genomics. 2025 Oct 6;26:883. doi: 10.1186/s12864-025-12132-5 (PMC12502372; doi:10.1186/s12864-025-12132-5)

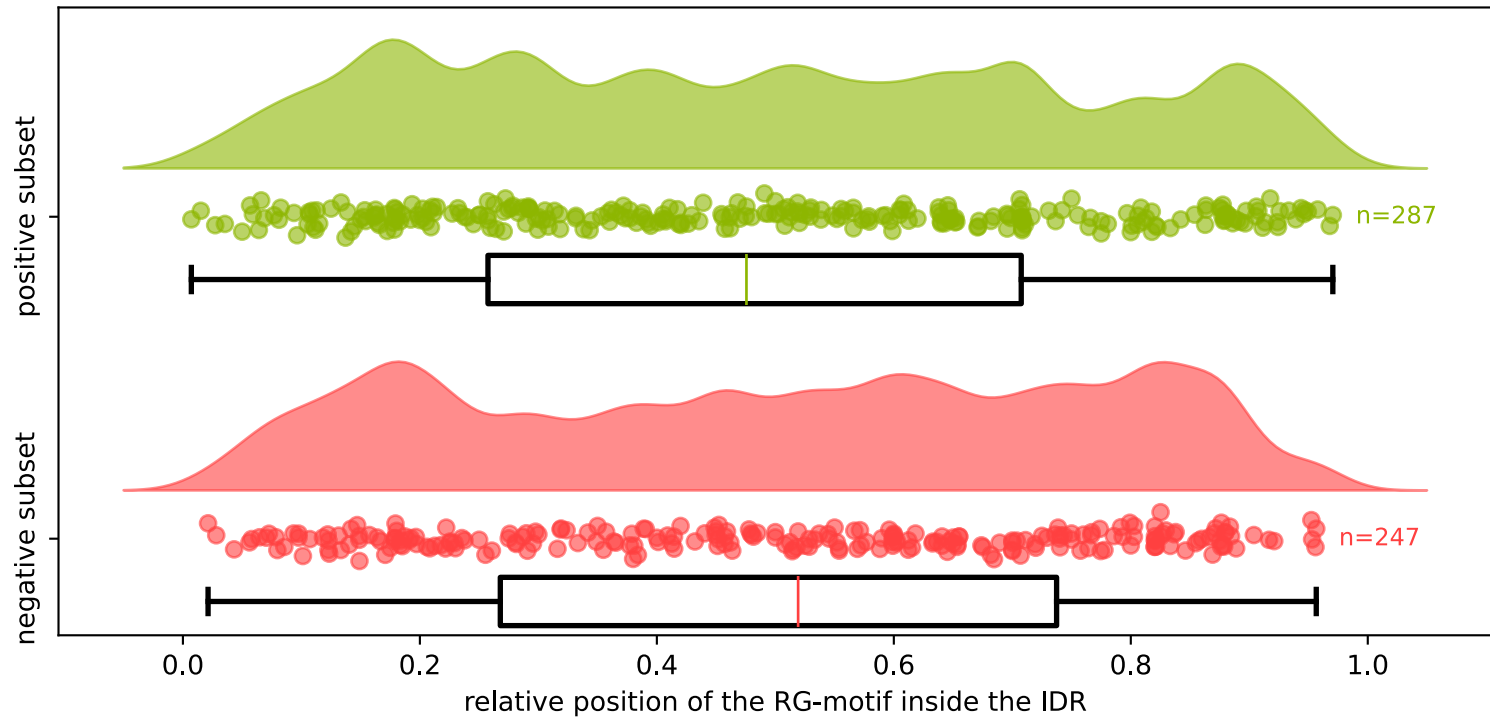

Supplement: Supplementary file 3 — Supplementary Material 3 [file 12864_2025_12132_MOESM3_ESM.pdf]

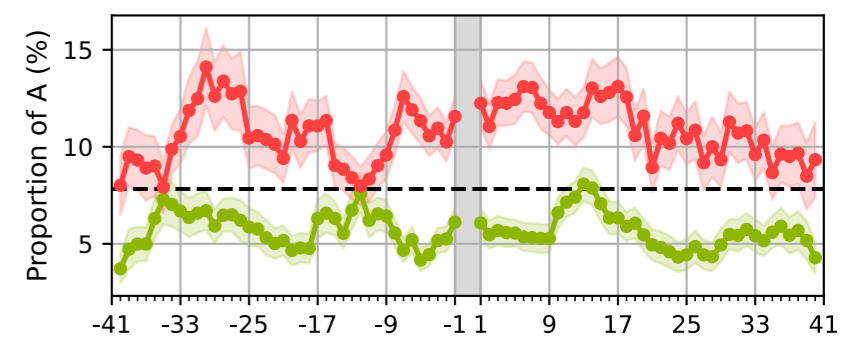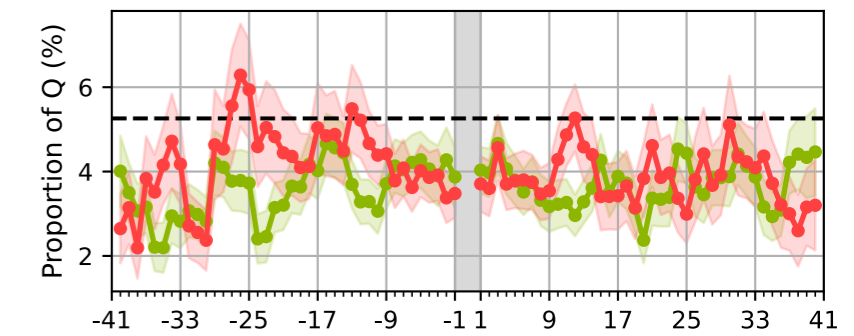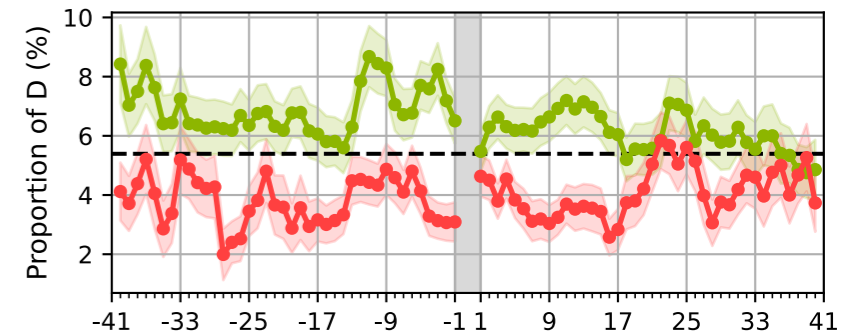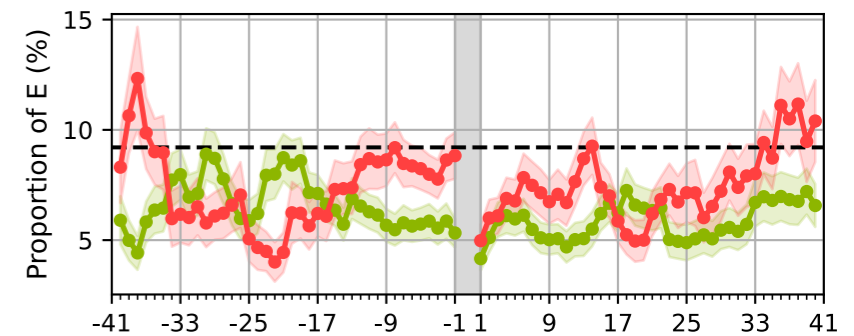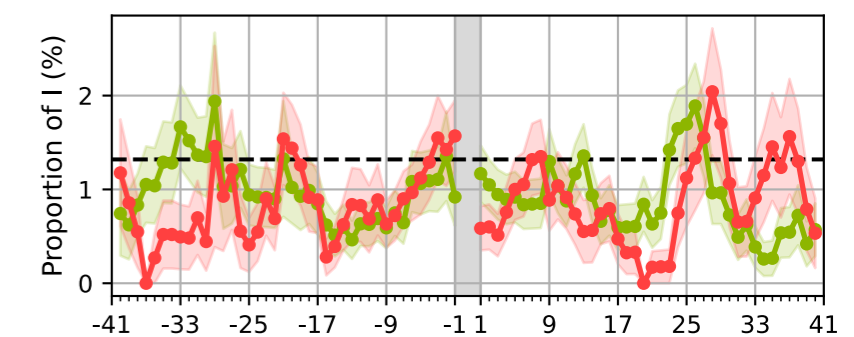

amino acid position relative to RG-motif

Supplement: Supplementary file 4 — Supplementary Material 4 [file 12864_2025_12132_MOESM4_ESM.pdf]
