# Supplementary material for: Computational investigation of the sequence context of arginine/glycine-rich motifs in the human proteome
Source: BMC Genomics. 2025 Oct 6;26:883. doi: 10.1186/s12864-025-12132-5 (PMC12502372; doi:10.1186/s12864-025-12132-5)
Supplement: Supplementary file 5 — Supplementary Material 5 [file 12864_2025_12132_MOESM5_ESM.pdf]

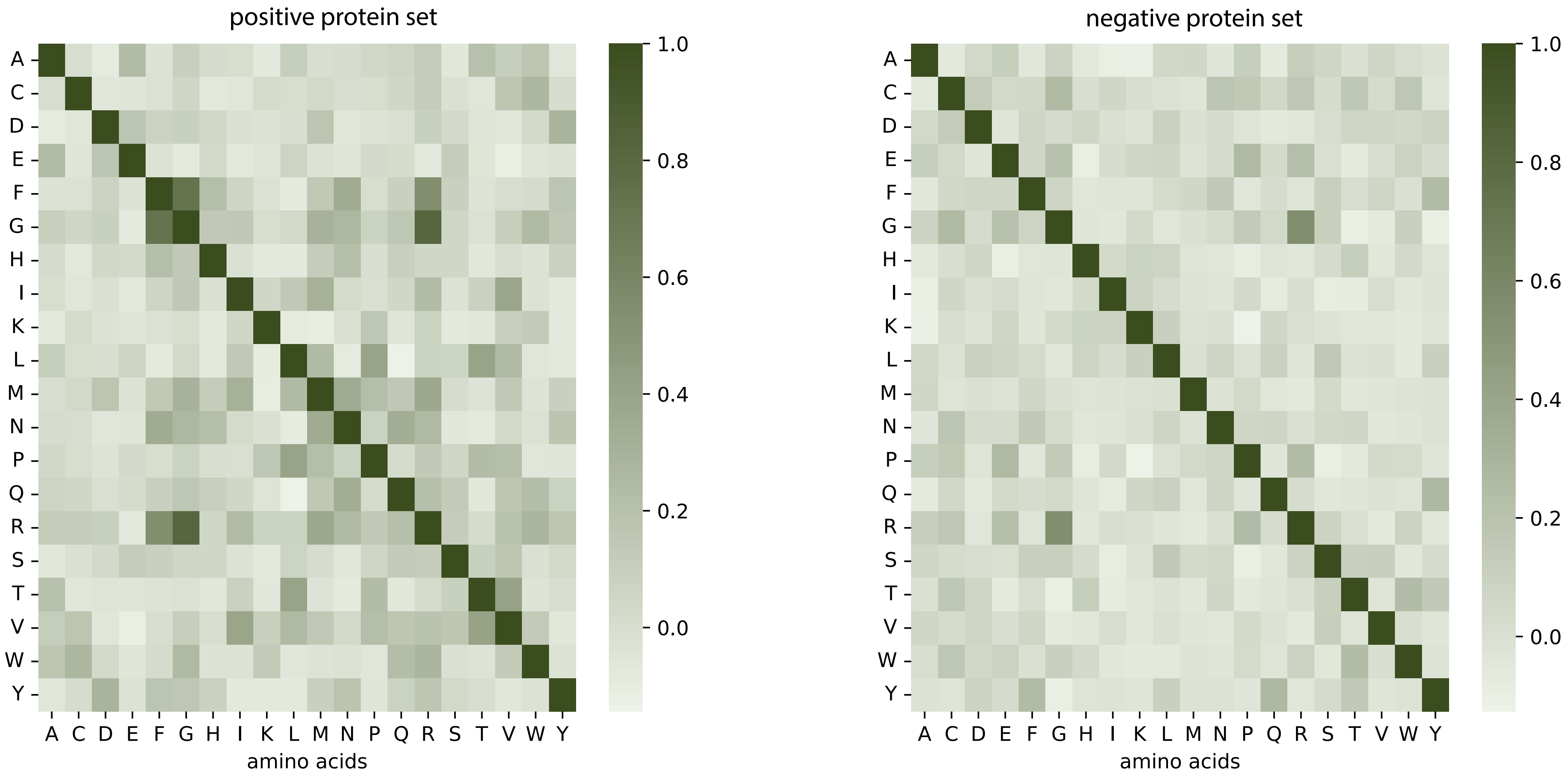

positive protein set
negative protein set
A C D E F G H I K L M N P Q R S T V W Y
amino acids
1.0
0.8
0.6
0.4
0.2
0.0
