# Supplementary material for: Computational investigation of the sequence context of arginine/glycine-rich motifs in the human proteome
Source: BMC Genomics. 2025 Oct 6;26:883. doi: 10.1186/s12864-025-12132-5 (PMC12502372; doi:10.1186/s12864-025-12132-5)
Supplement: Supplementary file 6 — Supplementary Material 6 [file 12864_2025_12132_MOESM6_ESM.docx]

Supplementary Material Captions:

**Computational investigation of the sequence context of arginine/glycine-rich motifs in the human proteome**

Eric Schumbera, Dorothee Dormann, Andreas Walther, Miguel A. Andrade-Navarro

**Supplementary material** S1: List of GO child terms for the GO term GO:0003676 (nucleic acid binding)

**Supplementary material** S2: Table of the human RG proteome (proteins that contain at least one RG motif), including their protein length, number of motifs, motif sequence, motif location and whether the protein is predicted to be involved in phase separation (LLPS_property) and whether the protein has at least one NA-binding GO term annotated (NA_binding_property). The motif sequence and the location of the motif are separated by a semicolon if multiple motifs exist in one protein.

**Supplementary material** S3: Comparison of the locations of the RG motifs within their IDRs between the positive and the negative subsets. A value of 0 means that the RG motif is located at the very N-terminal end of the IDR, whereas a value of 1 means that the RG motif is located at the C-terminal end of the IDR.

**Supplementary material** S4: Amino acid proportion distance plot for all amino acids between the positive and the negative subsets. For details, see the caption of Figure 4.

**Supplementary material** S5: Correlation matrices of the RG motifs for the positive and the negative subset between all amino acids.
